# Comparative study on the performance of different classification algorithms, combined with pre- and post-processing techniques to handle imbalanced data, in the diagnosis of adult patients with familial hypercholesterolemia

**DOI:** 10.1371/journal.pone.0269713

**Published:** 2022-06-24

**Authors:** João Albuquerque, Ana Margarida Medeiros, Ana Catarina Alves, Mafalda Bourbon, Marília Antunes

**Affiliations:** 1 Departamento de Biomedicina, Unidade de Bioquímica, Faculdade de Medicina, Universidade do Porto, Porto, Portugal; 2 Centro de Estatística e Aplicações, Faculdade de Ciências, Universidade de Lisboa, Lisboa, Portugal; 3 Grupo de Investigação Cardiovascular, Departamento de Promoção da Saúde e Prevenção de Doenças Não Transmissíveis, Instituto Nacional de Saúde Doutor Ricardo Jorge, Lisboa, Portugal; 4 Instituto de Biossistemas e Ciências Integrativas, Faculdade de Ciências, Universidade de Lisboa, Lisboa, Portugal; 5 Departamento de Estatística e Investigação Operacional, Faculdade de Ciências, Universidade de Lisboa, Lisboa, Portugal; University of the Aegean School of Social Sciences, GREECE

## Abstract

Familial Hypercholesterolemia (FH) is an inherited disorder of cholesterol metabolism. Current criteria for FH diagnosis, like Simon Broome (SB) criteria, lead to high false positive rates. The aim of this work was to explore alternative classification procedures for FH diagnosis, based on different biological and biochemical indicators. For this purpose, logistic regression (LR), naive Bayes classifier (NB), random forest (RF) and extreme gradient boosting (XGB) algorithms were combined with Synthetic Minority Oversampling Technique (SMOTE), or threshold adjustment by maximizing Youden index (YI), and compared. Data was tested through a 10 × 10 repeated *k*-fold cross validation design. The LR model presented an overall better performance, as assessed by the areas under the receiver operating characteristics (AUROC) and precision-recall (AUPRC) curves, and several operating characteristics (OC), regardless of the strategy to cope with class imbalance. When adopting either data processing technique, significantly higher accuracy (*Acc*), *G*-mean and *F*_1_ score values were found for all classification algorithms, compared to SB criteria (*p* < 0.01), revealing a more balanced predictive ability for both classes, and higher effectiveness in classifying FH patients. Adjustment of the cut-off values through pre or post-processing methods revealed a considerable gain in sensitivity (*Sens*) values (*p* < 0.01). Although the performance of pre and post-processing strategies was similar, SMOTE does not cause model’s parameters to loose interpretability. These results suggest a LR model combined with SMOTE can be an optimal approach to be used as a widespread screening tool.

## Introduction

Familial Hypercholesterolemia (FH) is an inherited disorder of lipid metabolism, characterized by increased low density lipoprotein cholesterol (LDLc) plasmatic concentrations [1]. If left untreated, the high cholesterol levels from birth lead to premature atherosclerosis, which constitutes a major risk factor for cardiovascular disease (CVD) [1, 2].

FH is caused by autosomal dominant mutations in one of three key encoding genes: *LDLR*, *APOB* or *PCSK9*. Pathogenic variants in the *LDLR* gene are the most common, accounting for around 90% of FH cases, while variants in *APOB* and *PCSK9* genes are found in around 5% and 1% of FH cases, respectively [3–5]. A very small percentage of FH cases is attributed to rare monogenic variants in a novelty gene involved in lipoprotein metabolism, or to a severe form of polygenic hypercholesterolemia [6]. According to inheritance pattern, FH can be classified as heterozygous (HeFH) or homozygous (HoFH). HeFH, hereinafter referred only as FH, is the most common form of the disease, affecting 1:200–500 individuals worldwide [7, 8]. Because signs and symptoms are less pronounced in this case, the disease can go undetected for several decades with severe, and often irreversible consequences. An early diagnosis of FH, followed by introduction of adequate therapeutic measures, is therefore considered of paramount importance [9].

Genetic diagnosis can provide a definite FH diagnostic [10], but is costly and time-consuming, thus a previous selection of patients to undergo molecular testing must be performed. Simon Broome (SB) criteria [11], are among the most commonly used clinical methods available for the diagnosis of FH, and are based on LDLc and total cholesterol (TC) levels, presence of tendon xanthomas and family history [6, 9]. Such criteria however present important limitations, when applied to the general population. The first of all relates to the classical dilemma of balance between sensitivity (*Sens*) and positive predictive value (*PPV*). In fact, for patients with possible or definite FH according to SB criteria, *Sens* was shown to be very high, varying from to 90–94%, but *PPV* was reduced, varying form 27–39% [12–14]. Results from these studies illustrate that these criteria are very conservative, with most mutation carriers identified, but with a high number of false positive cases also retained. Another limitation of SB criteria is that they require information on family history of hypercholesterolemia and premature CVD, which is often absent, restricting their applicability in clinical practice [15]. On the other hand, they do not make use of other potentially informative indicators routinely assessed in primary care settings, such as serum concentrations of additional biochemical parameters, or other biological and clinical variables. The development of classification algorithms incorporating such features can prove to be a simple and effective approach to identify patients with the highest risk of having FH.

### Classification algorithms and strategies to deal with imbalanced data

The classification problem in the current study deals with a binary dependent variable, representing the positive or negative diagnosis for FH. The FH diagnosis is estimated with basis on the relation with several categorical or quantitative predictor variables. There are several classification procedures available for this type of problem, based on different methodological approaches. In the current study, logistic regression (LR), naive Bayes classifier (NB), random forest (RF) and extreme gradient boosting (XGB) models have been tested.

LR is a classical statistical approach, and a special case of the generalized linear models (GLM) methodology [16]. NB is a probabilistic classifier based on Bayes’ theorem. The term “naive” arises from the assumption that predictor features are conditionally independent, which is not always accurate. Despite its simplicity, NB classifier has shown comparable performance even with highly sophisticated classification methods [17]. RF is a machine learning algorithm that aggregates the results of multiple individual classifiers, most commonly decision trees (DT), to classify an observation, and is therefore designated as an ensemble learning method [18]. Each DT is grown using a random bootstrapped sample of the training data, a method known as *bagging* [19], and only a random subset of predictor variables is tested in each of the trees, a process designated as the *random subspace selection method* [20]. By weighting the result over the many trees, the final outcome is less subject to random fluctuations in the training dataset, which should provide an increased capacity for generalising patterns [18]. The XGB model is also an ensemble learning algorithm, part of the boosted classifiers family. Simply put, XGB works by fitting a given classification model, calculating the model’s residuals, and fitting subsequent models on the estimated residuals [21]. For each iteration, optimization of the the loss function is performed following a gradient descent method [22, 23]. Unlike RF, the several DT that constitute XGB are not independent, but added sequentially, making the final algorithm a stagewise additive model [21].

The imbalanced data problem refers to a classification task where the number of observations in each class is not equally distributed. Furthermore, the observations that constitute the minority class, are generally the ones that represent the outcome of interest, and present higher misclassification costs [24, 25]. The biggest concern regarding this matter, is the fact learning algorithms are generally designed to minimize overall error, i.e., to achieve maximum predictive accuracy (*Acc*). In such a situation, the classification algorithm will place more emphasis on learning from data observations that occur more commonly, leading to higher misclassification rates for the minority class [24, 26, 27]. This is a common problem in many areas of research, and is given particular emphasis on the medical diagnosis field. In this case, the minority class is represented by the patients which in fact are FH, whereas non-FH patients represent the majority class.

Several techniques have been suggested to deal with the class imbalance problem, at different stages of the classification algorithm implementation process [24, 25, 27]. Interventions to deal with imbalanced data issues at a pre-processing level generally refer to the use of data sampling methods to balance class distribution [25]. One of the most cited approaches in this field is the oversampling method proposed by Chawla et al. [26], designed as SMOTE (*Synthetic Minority Over-sampling Technique*). According to SMOTE, observations in the dataset minority class are used to synthetically generate new data points, by interpolating between samples in the original dataset. Unlike a simple resampling process, the dataset variance is not artificially reduced by this method, inducing the classifier to generalize better [26]. Post-processing strategies to manage data imbalance issues typically refer to adjusting a suitable cut-off value for the classification task, after implementing the classification algorithm. Since the output obtained by a given classifier is generally a continuous value, taking the form of a certain support function, a class probability estimate, or number of votes with the more recent ensemble algorithms, adjusting a threshold on this output is a relatively simple task, although deciding on the cut-off value to adopt may not be as simple [24, 27]. Provost [28] has demonstrated that more complex methods to deal with imbalanced data do not necessarily perform better than adjusting the threshold value. On the other side, some limitations of threshold adjustment can be pointed out, such as the possibility of overdriving the classifier towards the minority class, thus largely increasing the error on the majority instances [28], or the fact that model’s interpretability becomes meaningless as it was obtained optimising a loss function that is not in accordance with the intended decision border [27].

### Selection of metrics to assess classification performance

The results obtained by a given classification algorithm applied to a two-class problem, are generally presented in a confusion matrix. The confusion matrix is a 2 × 2 contingency table which provides, for each class, the instances that were correctly and incorrectly classified, like shown in Table 1.

**Table 1:** Confusion matrix for a binary outcome (adapted from Fawcett [30]).

		**Predicted class**	
		Positive	Negative
**Actual class**	Positive	True positive (TP)	False Negative (FN)
	Negative	False Positive (FP)	True Negative (TN)

Based on the different possible outcomes, several operating characteristics (OC), can be calculated. Like mentioned before, *Acc* is the most frequently used metric to assess the performance of a given classification algorithm, but may not be the most suitable measure in an imbalanced domain. To achieve this goal, other measures, which quantify the classification performance on positive and negative classes independently, are proposed. Other commonly used metrics are sensitivity (*Sens*) and specificity (*Spec*), which respectively represent the proportion of subjects with the disease that present a positive test result, and the proportion of subjects without the disease that present a negative test result, and positive (*PPV*) and negative (*NPV*) predictive values, which represent the proportion of subjects with a true positive test among all those who tested positive for the disease, and the proportion of subjects with a true negative test among all those who tested negative.

To achieve good classification results for both classes poses a particularly difficult challenge, since some of these measures exhibit a trade-off, and it is impractical to simultaneously monitor several measures. Alternative metrics have been developed for this reason, such as the *G*-mean, the *F*_*β*_ score, or the area under the curve (AUC) from receiver operating characteristic (ROC) or precision-recall (PR) graphics.

*G*-mean is one of the most frequently used measures when dealing with an imbalanced dataset [29]. This metric is simply the geometric mean between *Sens* and *Spec*, calculated as

$$\begin{array}{c} G=\sqrt{Sens\times Spec}. \end{array}$$ (1)

*G*-mean is therefore an indicator of the balance between the majority and minority class error in a classification test. Another interesting performance metric commonly used is the *F*_*β*_ score [29], which combines both *Sens* and *PPV*, and is defined as

$$\begin{array}{c} F_{\beta }=\frac{(1+\beta^{2})\times PPV\times Sens}{\beta^{2}\times PPV+Sens}. \end{array}$$ (2)

*F*_*β*_ is more informative about the effectiveness of a classifier on correctly predicting the cases in the minority class. The *β* coefficient presented in the equation represents a constant to adjust the relative importance of *Sens* with respect to *PPV*. Specifically, if *β* = 1, *Sens* and *PPV* have the same weight, and this metric will correspond to the harmonic mean of the two OC.

A very popular metric in this field is the production of a ROC curve, a graphic of *Sens* versus 1 − *Spec* values over all possible cut-off values of the classifier [30]. This graphic allows the visualization of the relative trade-off between benefits (TP rate) and costs (FP rate) [27]. The area under the ROC curve (AUROC) is considered an overall measure of the test discriminatory ability. This measure has been extensively used to compare performance between different classification algorithms, and is suitable to use with imbalanced samples, since it takes the class distribution into consideration [24, 27]. The ROC curve is also a very important instrument to adjust the cut-off value for a given classifier. One of the most frequently used criteria to select the best possible threshold from ROC curve analysis, is to maximize *Sens* and *Spec* summation, also known as Youden index (*YI*), in the following way:

$$\begin{array}{c} YI_{max}=\underset{t}{\text{max}}\left\{Sens\left(t\right)+Spec\left(t\right)-1\right\}, \end{array}$$ (3)

where *t* denotes the threshold for which *YI* is maximal. Geometrically, this corresponds to the point in the ROC curve with higher vertical distance from the *y* = *x* diagonal line. This criteria attributes equal importance to *Sens* and *Spec* values, ignoring the relative size of both classes [29, 31, 32]. Another metric of particular relevance, specially when dealing with an imbalanced dataset, is the PR curve [33]. This curve is obtained by plotting the *PPV* (or precision), against *Sens* (or recall), across all possible cut-off values. The area under the PR curve (AUPRC) serves as an overall performance indicator of the model in classifying solely positive instances [34].

### Previous work

Previous studies implementing LR models for FH diagnosis have reported an overall good performance for this model in identifying FH cases [35, 36]. In a study conducted with a cohort from the Dutch FH screening programme, Besseling et al. [35] have reported an AUROC of 0.85 for a LR model, and an even higher AUROC of 0.95 in an external validation sample of subjects from an outpatient lipid clinic. In a large cohort study, Weng et al. [36] have developed a LR model (FAMCAT) using data from the UK Clinical Practice Research Datalink, obtaining a similar AUROC of 0.86 in the validation cohort. In a more recent external validation study, FAMCAT model presented an AUROC of 0.83, and significantly higher discriminatory ability than SB or DLCN clinical criteria [37]. Interesting results have also been obtained with the most recent use of ensemble learning algorithms. In a project supported by the FH Foundation, Banda et al. [38] have developed a RF algorithm named FIND FH, for which internal validation results showed a AUROC of 0.94, and a AUPRC of 0.71, with *PPV* = 0.88, *Sens* = 0.75 and *Spec* = 0.99. Pina et al. [39] have tested the performance of a gradient boosting machine (GBM) in two independent cohorts, with reported AUROC of 0.83 and 0.78, respectively. The GBM developed in this study revealed higher discriminatory ability than DLCN criteria and other machine learning algorithms, such as DT and a neural network. To the knowledge of the authors, there are no published studies assessing the performance of NB classifier for FH diagnosis.

Comparison of different classification algorithms applied to FH diagnosis is a relatively a scarce topic in the literature. In a recent paper however, Akyea et al. [40] have investigated the performance of an array of machine learning algorithms in identifying FH in a large cohort of more than 4 million patients in the UK, reporting an AUROC of 0.81 for a LR model, and a higher AUROC of 0.89 for RF and GBM models. In another study, Niehaus et al. [41] focused on comparing the performance of FIND FH with a LR model, and found improved AUROC (0.91 vs 0.82), *Sens* (0.61 vs 0.56) and *Spec* (0.96 vs 0.91) values for the RF model compared to the LR model.

The main purpose of this work was to explore alternative classification procedures for FH diagnosis, based on different biological and biochemical indicators, and compare their performance with SB traditional clinical criteria. The classification algorithms developed for this purpose were LR, NB, RF and XGB models. An optimal cut-off value for FH diagnosis was searched either by applying a SMOTE pre-processing method, or a post-processing technique, selecting the threshold which maximizes *YI*.

## Materials and methods

### Study sample and data

The sample used in the current study was taken from the Portuguese FH study, an ongoing study started in 1999 with the purpose of identifying and characterizing FH in the Portuguese population [42]. 513 observations, corresponding to index adult patients of both sexes (18–78 years of age), with TC and LDLc values over the 75^th^ percentile defined for the Portuguese population [43], which corresponds to TC ≥212 mg/dL or LDLc ≥123 mg/dL for women, or TC ≥216 mg/dL or LDLc ≥141 mg/dL for men, were initially retrieved. For patients undergoing lipid lowering therapy (LLT) at time of biochemical assessment, pre-medicated cholesterol values were used as inclusion criteria, or in case these were not available, estimated using the correction factors proposed by Benn et al. [44]. Patients undergoing other forms of LLT than statin therapy, prescribed either as monotherapy or combined with ezetimibe, as well as patients without information regarding LLT and with no previous cholesterol values, presenting a variant of unknown significance (VUS), a monogenic variant in a rare gene or HoFH, were excluded from the study. The final dataset was comprised of 451 individuals, *n* = 334 medicated patients (*n* = 111 with positive molecular diagnosis for FH), and *n* = 117 non-medicated patients (*n* = 35 with positive molecular diagnosis for FH). All subjects were white, of European ancestry. At time of assessment, participants were receiving standard healthcare and nutritional advice from the family physician at the hospital. Participants signed an informed consent, and information was registered in a confidential database, legalized by the National Data Protection Commission. The study complies with the Declaration of Helsinki and was approved by the ethics committee of the Instituto Nacional de Saúde Doutor Ricardo Jorge (INSA), in Lisbon.

Serum concentrations for a panel of several biochemical variables related to lipid metabolism were used as candidate predictor variables: TC, LDLc, high density lipoprotein cholesterol (HDLc), triglycerides (TG), apolipoproteins AI (ApoAI) and B (ApoB), and lipoprotein(a) (Lp(a)). Concentrations were determined in mg/dL, by enzymatic and colorimetric methods, using a Cobas Integra 400 Plus (Roche) analyzer [45]. Additional variables, regarding biological and clinical information, were also included. These variables were age, body mass index (BMI), presence of physical signs (corneal arcus before the age of 45, tendon xanthomas, xanthelasma), occurrence and age of a previous CVD event (defined as history of miocardial infarction, acute coronary syndrome, cerebrovascular accident, transient ischemic attack, peripheral artery disease, percutaneous transluminal coronary angioplasty or coronary artery bypass graf), hypertension, diabetes (type I or type II), hypothyroidism, and smoking and drinking habits. Other secondary causes of hypercholesterolaemia, such as liver disease (fatty liver disease, cirrhosis, chronic liver failure, alcoholic liver disease), kidney disease (chronic kidney disease, renal impairment, acute renal failure), and nephrotic syndrome [36], were excluded. Molecular diagnosis was performed by the study of the *LDLR*, *APOB* and *PCSK9* genes, through next-generation sequencing (NGS) [45]. Participants with a positive molecular diagnosis were classified as FH, and participants with a negative molecular diagnosis classified as non-FH.

### Comparison between classification algorithms combined with techniques to manage data imbalance

Exploratory analysis of biological and biochemical variables was initially performed for FH and non-FH subjects in both datasets. Non parametric Mann-Whitney-Wilcoxon test (MWW) was used to test for differences in continuous variables, given poor adjustment to normal distribution by some of these predictors, and chi-squared test was used to assess for differences in categorical predictor variables. Missing values were imputed in a two-step process. In a first step, LLT usage was imputed for patients possessing a lipid profile assessed in a previous moment, by means of a previously validated GLM model, using the percentage differences between two lipidic measures [46]. In a second step, other variables with missing values were imputed by means of *k*-nearest neighbours method using Gower’s dissimilarity measure [47]. Variables which required imputation presented a percentage of missing values between 1% and 14%, which under the correct assumptions, can be expected to provide unbiased results [48].

Feature selection for LR models was performed by purposeful selection methods, after assessing for collinearity through variance inflation factors (VIF) analysis. For NB models, all variables for which significant differences were found between FH and non-FH patients, and presenting low correlation values among each other (*r* ≤ 0.4) [49] were retained. For highly correlated variables, selection of the variable to retain was performed through AUROC comparison. Continuous variables were also log-transformed for the NB models, whenever a better adjustment to the Normal distribution was obtained. Search for optimal RF models was conducted iteratively, using out-of-bag (OOB) estimates for performance comparisons. In a first step, hyperparameter tuning was performed for *ntree*, *mtry* and *node size*. RF with *ntree* between 10 and 2000, by increments of 10 trees, *mtry* ranging from 1 to 7 variables, and *node size* = 1, 5, 10, 15 and 20 were explored. In a second step, less informative variables were sequentially excluded, from the initial set of candidate predictors. The RF model was developed and implemented using the *randomForest* package from R [50]. Optimal XGB parameter tuning was also performed by iterative procedures, using a grid-search built-in method, with a 5-fold cross validation (CV) design. Grid-search was conducted for *nrounds*, *max_depth*, *min_child_weight*, *colsample_bytree*, *eta*, *subsample*, and *gamma*. The XGB model was developed and implemented using the *rgboost* package from R [21].

Except for the LR algorithm, which can incorporate LLT as a covariate in the model, a two-branch training model was defined for the other classification algorithms, considering medicated and non-medicated observations, with testing observations posteriorly integrated in a single sample, according to estimated probabilities by either branch. Such division was made because use of this variable violates the assumption of independence in NB, and tree-based classifiers must separate medicated from non-medicated patients at the root node of every tree, in order for cut-off values of predictor variables in subsequent nodes to make sense.

Two different techniques were adopted to cope with data imbalance issues. As a pre-processing technique, SMOTE oversampling method was used to balance the number of observations in FH positive class [26]. New synthetic data was generated considering *k* = 5 nearest neighbours, using Gower similarity measure. Proportion of FH patients relatively to non-FH subjects was around 1:2, both for medicated and non-medicated samples. As a post-processing technique, a new cut-off value based on *YI* maximization was calculated. Both of these techniques were tested against the default cut-off *c* = 0.5, obtained from original data. For all classification models, combined with the different pre- and post-processing techniques, different OC were calculated: *Acc*, *Sens*, *Spec*, *PPV*, *NPV*, *G*-mean and *F*_1_ score [30, 51].

Model comparison was performed by means of repeated 10-fold CV, sampling 10 random replicas of the dataset. For each of the CV samples, the different classification models were fitted to the training sample, and then used to classify the testing observations. AUROC and AUPRC values were obtained for testing observations in each of the 10 dataset replicas, both for datasets using only original data and SMOTE data. Mean values of different OC were calculated for each fold, among each dataset replica, and compared using the corrected resampled t-test proposed by Nadeau & Bengio [54]. All models were also compared with adapted SB criteria for possible or definite FH, defined as TC >290 mg/dL or LDLc >190 mg/dL, plus one of the following criteria: physical signs, or history of premature CVD (before age 60 if female, or before age 55 if male) in the patient, or in a first or second degree relative, or family history of hypercholesterolemia (TC >290 mg/dL in an adult first or second degree relative, or TC >260 mg/dL in a child or sibling under 16 years of age). Statistical analysis was performed using R and R Studio software, adopting a significance level of *α* = 0.05, and using Bonferroni method to correct for multiple testing.

## Results

### Descriptive statistics

Descriptive statistics for biochemical and biological variables regarding FH and non-FH patients, in medicated and non-medicated datasets, are presented in Table 2. The number and percentage of missing values, which have been imputed, are presented in supporting information (S1 Table).

**Table 2:** Comparison of biological and biochemical values between FH and non-FH patients, according to statins usage.

	Medicated patients			non-Medicated patients		
	FH	non-FH	p-value	FH	non-FH	p-value
n (%)	111 (33.2)	223 (66.8)	-	35 (30.0)	82 (70.0)	-
Gene: n (%)						
LDLR	104 (93.7)	-	-	31 (88.6)	-	-
APOB	5 (4.5)	-	-	3 (8.6)	-	-
PCSK9	2 (1.8)	-	-	1 (2.8)	-	-
Male: n (%)	47 (42.3)	107 (48.0)	0.39	12 (34.3)	40 (48.8)	0.21
Age: mean (sd)	47.3 (14.8)	48.2 (13.0)	0.55	33.7 (12.2)	39.7 (10.8)	<0.01
BMI: mean (sd)	25.9 (4.2)	26.3 (3.9)	0.16	25.5 (4.7)	23.9 (3.4)	0.15
Physical signs: n (%)	27 (24.3)	19 (8.5)	<0.01	5 (14.3)	5 (6.1)	0.28
CVD disease: n (%)	32 (28.8)	74 (33.2)	0.50	6 (17.1)	9 (11.0)	0.54
Age CVD: mean (sd)	45.9 (11.8)	47.2 (9.8)	0.64	42.5 (10.3)	34.1 (6.7)	0.22
Hypertension: n (%)	39 (35.1)	62 (27.8)	0.62	4 (11.4)	6 (7.3)	0.71
Smoking: n (%)	16 (14.4)	49 (22.0)	0.13	5 (14.3)	23 (28.0)	0.17
Cigarettes/day: mean (sd)	11.8 (9.8)	13.5 (7.9)	0.29	14.0 (8.0)	12.5 (8.2)	0.72
Alcohol use: n (%)	16 (14.4)	49 (22.0)	0.99	4 (11.4)	24 (29.3)	0.07
Alcohol units/week: mean (sd)	10.3 (10.4)	10.0 (8.7)	0.67	2.5 (3.0)	6.4 (6.8)	0.14
Lipid profile (in mg/ dL)						
TC: mean (sd)	254.0 (58.0)	209.0 (46.0)	<0.01	335.0 (75.0)	279.0 (45.0)	<0.01
LDLc: mean (sd)	176.2 (52.6)	127.5 (41.0)	<0.01	256.7 (70.3)	195.2 (41.1)	<0.01
HDLc: mean (sd)	55.2 (15.9)	56.7 (16.1)	0.31	52.7 (17.5)	57.1 (17.5)	0.17
TG: mean (sd)	116.3 (57.1)	141.5 (74.5)	<0.01	123.9 (51.1)	141.2 (61.6)	0.23
Lp(a): mean (sd)	59.0 (56.2)	59.7 (63.5)	0.74	42.2 (60.7)	42.7 (52.7)	0.61
ApoAI: mean (sd)	151.0 (36.0)	161.0 (35.0)	<0.01	146.0 (39.0)	162.0 (40.0)	0.05
ApoB: mean (sd)	132.4 (44.9)	99.5 (31.4)	<0.01	179.9 (38.3)	136.4 (36.1)	<0.01

FH: familial hypercholesterolemia; BMI: body mass index; CVD: cardiovascular disease; TC: total cholesterol; LDLc: low density lipoprotein cholesterol; HDLc: high density lipoprotein cholesterol; TG: triglycerides; Lp(a): lipoprotein(a); Apo: apolipoprotein.

### AUROC and AUPRC values

**Fig 1:**
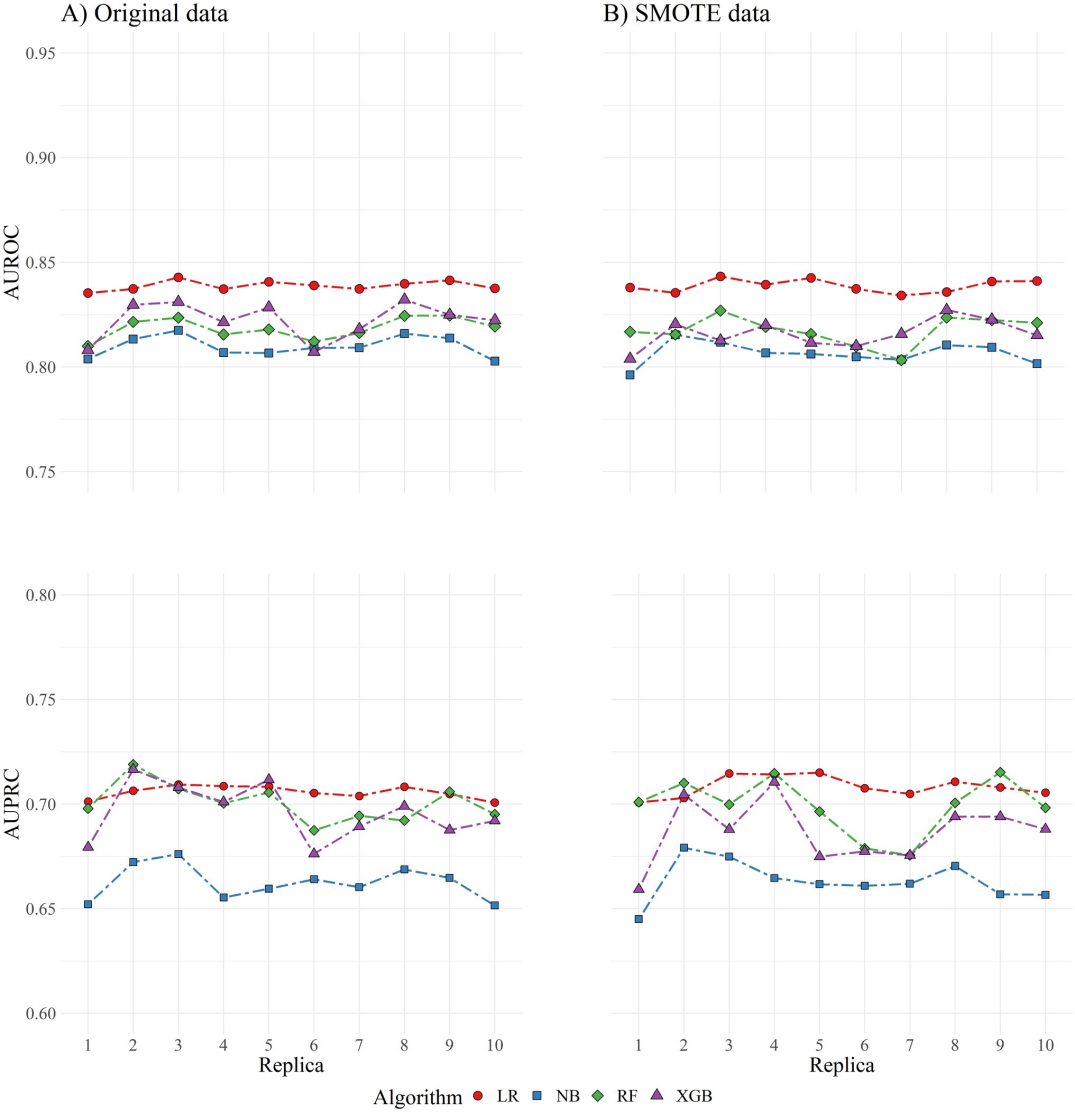
Comparison of areas under the ROC and PR curves, for each replica of the dataset. On the left column are presented the results using original sample data, and on the right column the results using SMOTE sample data. AUROC: area under the receiver operating characteristics curve; AUPRC: area under the precision-recall curve; LR: logistic regression; NB: naive bayes; RF: random forest; XGB: extreme gradient boosting; SMOTE: synthetic minority oversampling technique.

The AUROC and AUPRC for medicated and non-medicated datasets, as obtained in testing sets from every sampled replica of the dataset, concerning original and SMOTE data, are presented in Table 3 and Fig 1. Global estimates for AUROC and AUPRC, calculated from the combined probability estimates of all testing observations, are also presented in Table 3.

**Table 3:** Area under the ROC and PR curves for medicated patients, using the original and SMOTE sample data.

Model	*m*_1_	*m*_2_	*m*_3_	*m*_4_	*m*_5_	*m*_6_	*m*_7_	*m*_8_	*m*_9_	*m*_10_	*M*
**AUROC original data**											
LR	0.84	0.84	0.84	0.84	0.84	0.84	0.84	0.84	0.84	0.84	**0.84**
NB	0.80	0.81	0.82	0.81	0.81	0.81	0.81	0.82	0.81	0.80	**0.81**
RF	0.81	0.82	0.82	0.82	0.82	0.81	0.82	0.82	0.82	0.82	**0.82**
XGB	0.81	0.83	0.83	0.82	0.83	0.81	0.82	0.83	0.82	0.82	**0.82**
**AUROC SMOTE data**											
LR	0.84	0.84	0.84	0.84	0.84	0.84	0.83	0.84	0.84	0.84	**0.84**
NB	0.80	0.82	0.81	0.81	0.81	0.80	0.80	0.81	0.81	0.80	**0.81**
RF	0.82	0.82	0.83	0.82	0.82	0.81	0.80	0.82	0.82	0.82	**0.82**
XGB	0.80	0.82	0.81	0.82	0.81	0.81	0.82	0.83	0.82	0.82	**0.82**
**AUPRC original data**											
LR	0.70	0.71	0.71	0.71	0.71	0.71	0.70	0.71	0.70	0.70	**0.71**
NB	0.65	0.67	0.68	0.66	0.66	0.66	0.66	0.67	0.66	0.65	**0.66**
RF	0.70	0.72	0.71	0.70	0.71	0.69	0.69	0.69	0.71	0.70	**0.70**
XGB	0.68	0.72	0.71	0.70	0.71	0.68	0.69	0.70	0.69	0.69	**0.70**
**AUPRC SMOTE data**											
LR	0.70	0.70	0.71	0.71	0.72	0.71	0.70	0.71	0.71	0.71	**0.71**
NB	0.65	0.68	0.67	0.66	0.66	0.66	0.66	0.67	0.66	0.66	**0.66**
RF	0.70	0.71	0.70	0.71	0.70	0.68	0.68	0.70	0.72	0.70	**0.70**
XGB	0.66	0.70	0.69	0.71	0.67	0.68	0.68	0.69	0.69	0.69	**0.69**

AUROC: area under the receiver operating characteristics curve; AUPRC: area under the precision-recall curve; LR: logistic regression; NB: naive bayes; RF: random forest; XGB: extreme gradient boosting; SMOTE: synthetic minority oversampling technique; Each different replica from the dataset is represented by *m*_*n*_, and *M* represents the combination of estimates obtained for observations in every replica.

### Operating characteristics comparison

**Fig 2:**
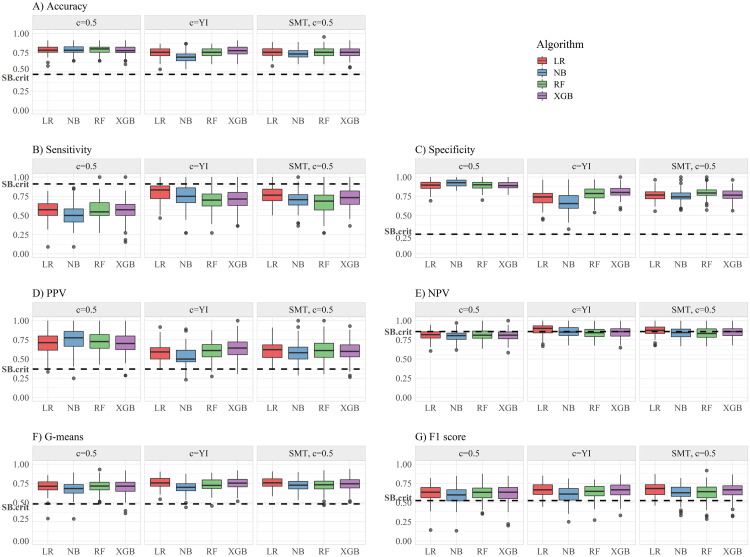
Comparison of operating characteristics values between different classification algorithms, and strategies to deal with data imbalance. The dashed line represents the value obtained when applying SB criteria. *Acc*: accuracy; *Sens*: sensitivity; *Spec*: specificity; *PPV*: positive predictive value; *NPV*: negative predictive value; SMT: synthetic minority oversampling technique; YI: Youden Index; LR: logistic regression; NB: naive Bayes; RF: random forest; XGB: extreme gradient boosting; SB: Simon Broome criteria.

The mean values of selected OC, obtained when applying different classification algorithms, combined with different strategies to cope with data imbalance, together with OC values obatained when applying SB diagnosis criteria, are presented in Table 4. Significant differences for the OC values between the different classification algorithms among a same data processing method, between these and SB criteria, and between different strategies to manage data imbalance among each classifier, are presented in Table 5, respectively. The distribution of the obtained OC for each classification method is presented in Fig 2.

**Table 4:** Mean and standard deviation values of operating characteristics (OC), for different classification algorithms and techniques to cope with data imbalance, and values obtained with SB criteria.

Model	*Acc*	*Sens*	*Spec*	*PPV*	*NPV*	*G*-mean	*F*_1_ score
***c* = 0.5, original dataset**							
LR	0.79 (0.06)	0.57 (0.12)	0.89 (0.06)	0.72 (0.14)	0.81 (0.07)	0.71 (0.08)	0.63 (0.10)
NB	0.79 (0.06)	0.51 (0.13)	0.92 (0.04)	0.75 (0.15)	0.80 (0.07)	0.68 (0.09)	0.60 (0.12)
RF	0.79 (0.06)	0.57 (0.13)	0.89 (0.06)	0.72 (0.13)	0.81 (0.07)	0.71 (0.08)	0.63 (0.10)
XGB	0.78 (0.06)	0.57 (0.14)	0.89 (0.05)	0.71 (0.14)	0.81 (0.07)	0.71 (0.09)	0.62 (0.12)
***c* = *YI*, original dataset**							
LR	0.75 (0.06)	0.81 (0.12)	0.72 (0.09)	0.58 (0.10)	0.89 (0.07)	0.76 (0.07)	0.67 (0.09)
NB	0.70 (0.07)	0.75 (0.15)	0.67 (0.12)	0.53 (0.12)	0.85 (0.08)	0.70 (0.08)	0.60 (0.11)
RF	0.75 (0.07)	0.69 (0.13)	0.78 (0.09)	0.60 (0.13)	0.84 (0.07)	0.73 (0.08)	0.64 (0.10)
XGB	0.77 (0.06)	0.71 (0.13)	0.81 (0.07)	0.64 (0.13)	0.85 (0.07)	0.75 (0.08)	0.66 (0.10)
***c* = 0.5, SMOTE dataset**							
LR	0.76 (0.06)	0.76 (0.11)	0.76 (0.08)	0.61 (0.11)	0.87 (0.07)	0.76 (0.07)	0.67 (0.09)
NB	0.73 (0.06)	0.71 (0.12)	0.75 (0.08)	0.58 (0.13)	0.84 (0.07)	0.72 (0.07)	0.63 (0.10)
RF	0.76 (0.07)	0.67 (0.13)	0.80 (0.07)	0.61 (0.13)	0.83 (0.07)	0.73 (0.09)	0.63 (0.11)
XGB	0.75 (0.07)	0.73 (0.13)	0.77 (0.07)	0.60 (0.12)	0.85 (0.07)	0.75 (0.08)	0.65 (0.11)
SB	0.47	0.91	0.26	0.37	0.86	0.48	0.53

*Acc*: accuracy; *Sens*: sensitivity; *Spec*: specificity; *PPV*: positive predictive value; *NPV*: negative predictive value; SMOTE: synthetic minority oversampling technique; LR: logistic regression; NB: naive Bayes; RF: random forest; XGB: extreme gradient boosting; SB: Simon Broome criteria.

**Table 5:** Significant differences for operating characteristics (OC) values among several classification methods.

**5.1:** Significant differences for operating OC values between different classification algorithms.							
Model	*Acc*	*Sens*	*Spec*	*PPV*	*NPV*	*G*-mean	*F*_1_ score
***c* = *YI*, original dataset**							
LR-NB	0.04↑	-	-	-	-	0.04↑	0.04↑
LR-RF	-	0.02↑	-	-	-	-	-
LR-XGB	-	0.04↑	0.02↓	-	-	-	-
NB-RF	0.01↓	-	0.01↓*	0.02↓	-	-	-
NB-XGB	<0.01↓*	-	<0.01↓*	<0.01↓*	-	0.01↓	0.04↓
***c* = 0.5, SMOTE dataset**							
LR-RF	0.03↑	-	0.04↑	-	-	-	-
NB-RF	-	-	0.04↓	-	-	-	-
**5.2:** Significant differences for OC values between SB criteria and classification algorithms.							
Model	*Acc*	*Sens*	*Spec*	*PPV*	*NPV*	*G*-mean	*F*_1_ score
***c* = 0.5, original dataset**							
SB-LR	<0.01↓*	<0.02↑*	<0.01↓*	<0.01↓*	-	<0.01↓*	0.01↓*
SB-NB	<0.01↓*	<0.01↑*	<0.01↓*	<0.01↓*	0.01↑	<0.01↓*	-
SB-RF	<0.01↓*	<0.01↑*	<0.01↓*	<0.01↓*	-	<0.01↓*	0.01↓*
SB-XGB	<0.01↓*	<0.01↑*	<0.01↓*	<0.01↓*	-	<0.01↓*	0.02↓
***c* = *YI*, original dataset**							
SB-LR	<0.01↓*	0.01↑	<0.01↓*	<0.01↓*	-	<0.01↓*	<0.01↓*
SB-NB	<0.01↓*	<0.01↑*	<0.01↓*	<0.01↓*	-	<0.01↓*	0.04↓
SB-RF/XGB	<0.01↓*	<0.01↑*	<0.01↓*	<0.01↓*	-	<0.01↓*	<0.01↓*
***c* = 0.5, SMOTE dataset**							
SB-LR/XGB	<0.01↓*	<0.01↑*	<0.01↓*	<0.01↓*	-	<0.01↓*	<0.01↓*
SB-NB/RF	<0.01↓*	<0.01↑*	<0.01↓*	<0.01↓*	-	<0.01↓*	0.01↓*
**5.3:** Significant differences for OC values between data processing methods.							
Model	*Acc*	*Sens*	*Spec*	*PPV*	*NPV*	*G*-mean	*F*_1_ score
**LR**							
0.5—*YI*	-	<0.01↓*	<0.01↑*	<0.01↑*	<0.01↓*	-	-
0.5—*SMT*	-	<0.01↓*	<0.01↑*	<0.01↑*	<0.01↓*	-	-
*YI*—*SMT*	-	-	0.03↓	-	-	-	-
**NB**							
0.5—*YI*	<0.01↑*	<0.01↓*	<0.01 ↑ *	<0.01↑*	<0.01↓*	-	-
0.5—*SMT*	0.02↑	<0.01↓*	<0.01↑*	<0.01↑*	<0.01↓*	-	-
*YI*—*SMT*	0.04↑	-	<0.01↓*	0.04↓	-	-	-
**RF**							
0.5—*YI*	-	<0.01↓*	<0.01↑*	<0.01↑*	0.01↓*	-	-
0.5—*SMT*	-	<0.01↓*	<0.01↑*	<0.01↑*	-	-	-
**XGB**							
0.5—*YI*	-	<0.01↓*	<0.01↑*	0.01↑*	0.01↓*	0.04↓	-
0.5—*SMT*	-	<0.01↓*	<0.01↑*	<0.01↑*	0.01↓*	-	-
*YI*—*SMT*	-	-	0.04↑	-	-	-	-

Significant differences are signalled with an ↑ or ↓, depending on whether the first model performs better or worst than the second. If *, differences are still significant after applying Bonferroni correction. If -, non-significant for *p* < 0.05; *Acc*: accuracy; *Sens*: sensitivity; *Spec*: specificity; *PPV*: positive predictive value; *NPV*: negative predictive value; SMOTE: synthetic minority oversampling technique; YI: Youden Index; LR: logistic regression; NB: naive Bayes; RF: random forest; XGB: extreme gradient boosting; SB: Simon Broome criteria. Non-reported pairwise comparisons did not present any significant difference.

## Discussion

The current work combined several classification algorithms with data processing techniques, in order to search for the best method to classify FH patients in a cohort of dyslipidemic adult subjects. The different models were compared both with molecular diagnosis and SB biochemical criteria. The molecular diagnosis of FH was considered as the gold standard, due to the biological definition of the disease, in which FH is characterized as a genetic disorder of lipid metabolism [6], corroborated by evidence from large cohort studies, in which individuals with clinical criteria for FH that are confirmed to have a causative pathogenic variant present a significant increase in the risk of CVD, when compared to clinical FH patients in whom a causative variant is not found [52].

Except for LR algorithm, which can incorporate LLT variable directly in the model, a two-branch training model was implemented for the other classifiers, with separate models built for medicated and non-medicated patients, and testing observations classified according to the appropriate branch. This was done since LLT is a confounding factor, by altering the serum concentration of lipidic parameters, which violates the independence assumption required for NB model, and prevents tree-based learning ensembles like RF and XGB of dividing the sample according to biochemical variables, without accounting for this factor. This procedure was preferred relatively to using pre-medication lipid concentrations for medicated patients, or estimating them using available correction factors, since pre-medication values are missing in many cases, and correction factors for statin usage only adjust for LDLc concentrations, not accounting for the potential effect of LLT on other biochemical markers.

An analysis of the AUROC and AUPRC, using both original and SMOTE training samples, was initially performed. The probability values for the construction of these curves were obtained for the testing samples in each of the 10-folds, and combined for each of the 10 sampled replicas of the dataset. Estimates from every replica were further combined to obtain a global performance measure. For both curves, the LR model revealed the the best performance, followed by RF and XGB models with similar performance, and NB model performing noticeably worst. The performance of LR model also seems to be more stable across dataset replicas, compared to the other two classifiers. Results were similar for original and SMOTE samples. The range of values found for the AUROC (0.81–0.84) and AUPRC (0.66–0.71) is generally comprised in the values presented by other authors [35–39]. However, unlike other studies [40, 41], higher discriminatory ability was generally found for LR model, which may be related to several factors. While the previously mentioned studies have included a vast number of predictor variables readily available in primary care records, in the current work, fewer and more specific biochemical parameters, were included. By decreasing the variability of the constituent trees in learning ensembles such as RF or XGB, the overall performance of such models may diminish compared to classical statistical models like LR. The referred studies are also mostly applied to electronic health records (EHR) of patients in the general population, and not with specific lipid traits, like in the current case. Additionally, the LR algorithm is, from the investigated models, the only one which accommodates for possible interactions among predictor variables, which may be significant in the present problem. The number of variables included in the LR model, including interactions, allowed fulfilling, for all cross-validation samples, the condition of having at least 10 events per variable (EPV), previously referred to provide unbiased estimates for the regression coefficients. [53].

In a second step of this work, specific cut-off values were calculated for each of the classification algorithms, and selected OC calculated, and compared with the values obtained using SB criteria for FH diagnosis. The definition of an optimal cut-off value is important, since it makes it possible to propose clear thresholds to consider a diagnosis of FH, and to directly compare the results obtained by different classification algorithms with the established clinical criteria. The selected cut-off values were the default *c* = 0.5 obtained with original data, a post-processing method of threshold adjustment, calculated according to the maximization of Youden index (*c* = *YI*), and a *c* = 0.5 applied after implementing SMOTE, a pre-processing oversampling technique. Both pre and post-processing methods aim to solve the problem of imbalanced data, by assigning equal importance to the classification error of FH positive instances, which are under represented in the sample. The results concerning the different OC were compared by means of the corrected resampled t-test, which accounts for the increased probability of committing a type I error, that occurs by violating the assumption of independence between observations, in a repeated *k*-fold testing design [54].

When comparing the distinct classification algorithms, significant differences only occur when data processing methods are applied, in particular when adjusting the cut-off value by maximizing *YI*. In this scenario, NB seems to be the model which reveals the worst balance across all OC. In fact, this model suffered great loss in terms of *Acc*, *Spec* and *PPV* compared with the other models (*p* < 0.05), suggesting that shifting the classifier towards the minority class will result in a worst performance in classifying negative instances correctly. Also, LR showed significantly higher *Sens* levels than RF and XGB, and lower *Spec* than XGB (*p* < 0.05). When using SMOTE method, significant differences are only found for RF, presenting lower *Acc* and *Spec* values than LR, and higher *Spec* values than NB (*p* < 0.05). Because the current work attempts to model an ideal cut-off value to optimize *Sens* and *Spec* values, a direct comparison of the obtained OC values with other studies is made difficult. Nevertheless, when considering only the default *c* = 0.5, the found OC values for the LR model are either in line [41], or somewhat improved [40], relatively to the ones reported by previous studies, whereas machine learning ensemble models values are generally lower [38, 40, 41], for motives already explored.

When compared to SB biochemical criteria, differences generally become much more pronounced. All classification models presented significantly lower *Sens* (*p* < 0.05), but higher values of *Acc*, *Spec*, *PPV* and *G*-means (*p* < 0.01), and in most cases significantly higher *F*_1_ score (*p* < 0.05), across the three data processing methods. It is important to note that the elevated *Sens* values in SB criteria are essentially due to very conservative cut-off values, which results in a high number of FP cases [9]. Significantly higher values shown by other classification methods, regarding metrics that ally *Sens* with *Spec*, in the case of *G*-mean, or with *PPV*, in the case of *F*_1_ score, suggest reducing the number of retained FP cases, while still maintaining a high true positive rate, may benefit the overall performance of the diagnosis algorithm. Moreover, reducing the number of potential candidates to undergo the molecular diagnosis, may have important repercussions in terms of the process cost-effectiveness. The obtained OC values with the application of SB criteria are comparable to the ones reported in previous work [12–14].

Finally, differences in OC values across the several data processing methods, for a same classification algorithm, were calculated. From the observation of these results, it becomes evident that adjusting for data imbalance issues, either by thresholding or oversampling methods, substantially improves *Sens* and *NPV*, while decreasing *Spec* and *PPV* (*p* < 0.01). This means that it is possible to greatly improve the correct classification rates of FH patients, with a corresponding increase in the misclassification rates of non-FH patients. NB was the only model that revealed significant differences on *Acc* levels, with losses from the original data *c* = 0.5 cut-off to *c* = *YI* and SMOTE data model (*p* < 0.05). Additionally, NB model revealed significant worst *Spec* and *PPV* values with the *YI* cut-off, compared to SMOTE processing technique (*p* < 0.05). These results seem to confirm that NB may be the most unstable model when applied together with a data processing method, particularly when attempting to adjust a cut-off value at the post-processing stage. No significant differences were found for *G*-means and *F*_1_ score according to data processing method. A potential advantage of using SMOTE pre-processing technique is the fact that model parameters and outcomes in the SMOTE model do not loose interpretability. For example, it would be much easier for a practitioner to understand that a given algorithm diagnoses a patient as FH positive because the predicted probability of having the disease is *p* = 0.6, as obtained from a SMOTE model, than *p* = 0.35, as obtained by adjusting the threshold according to *c* = *YI* to a model constructed using original data.

Important to note, the most relevant variables retained by the different classification algorithms are generally the same. As expected, LDLc is the most important predictor variable for all classification algorithms, independently of being medicated or not. For learning ensembles, which can incorporate highly correlated variables, ApoB and TC are also very important. Other biochemical variables, like ApoAI and/or HDLc, and TG, or clinical and biological indicators, such as Physical Signs and Age are also consistently important. Some of the predictor variables, such as Lpa, Gender, BMI, CVD, Hypertension or Alcohol Use, seem to be relevant only for some models, and dependent if the patient is on LLT. A thorough interpretation on the biological importance of these variables, including an analysis of the relationship between biochemical markers in lipoprotein metabolic pathway, will be addressed in a separate paper.

In summary, LR was considered the most robust and parsimonious model in the current problem. Independently of the chosen classification algorithm, adjustment of the cut-off values through pre or post-processing methods largely improves the retention of FH cases, with a modest decrease in *Acc*, and increase in *G*-mean and *F*_1_ score values. Although the performance of both strategies is similar, SMOTE technique improves the model interpretability. When compared to SB criteria, metrics which combine misclassification rates of positive and negative instances, specifically *Acc*, *G*-mean and *F*_1_ score, improve significantly. The final model fit for the LR model, as obtained in combination with SMOTE technique, is presented in supporting information (S2 Table). A pilot internet application, named FH.ID.Tool, was developed using R Studio Shiny package, and can be consulted at https://fhidtool.shinyapps.io/dyslipid/. Physicians that collaborate with the Portuguese FH study will be invited to test this application and provide feedback on how to improve its functionality to a final version, that will be proposed for implementation at the national health system.

## Conclusion

Several conclusions can be taken from the current study. The LR model was the one which presented higher AUROC and AUPRC values, both for the original and SMOTE datasets, while NB presented the lowest values. When comparing OC values for specifically selected cut-off points, resulting from the implementation of several classification algorithms combined with different data processing methods, LR kept an overall robust performance, while NB presented the most imbalanced metrics, particularly when adjusting the cut-off value by maximizing *YI*. Differences become much more pronounced when comparing the several classification algorithms with SB criteria for FH diagnosis. All classification models presented significantly higher *Acc*, *Spec*, *PPV* and *G*-means, and in most cases significantly higher *F*_1_ score than SB criteria (*p* < 0.01), across all data processing methods. Higher *Sens* values obtained with SB criteria are obtained at the expense of high FP retention, revealing poor discriminatory ability. Based on the differences in OC values between different strategies to adjust the cut-off value, for a same classification algorithm, *Sens* and *NPV* seem to improve greatly with use of pre or post-processing techniques, with the inverse tendency verified for *Spec* and *PPV*. A non-significant loss in *Acc* is attributed to the incorrect classification of non-FH patients, while other metrics that attribute the same weight to positive and negative error rates, *G*-means and *F*_1_ score, seem to benefit from data processing strategies. The results obtained using *c* = *YI* with original data, or applying SMOTE method, were similar, with SMOTE method presenting the advantage of maintaining the interpretability of the model parameters. For the presented problem, the use of a LR model, combined with SMOTE pre-processing technique, is the proposed method for more parsimonious and interpretable results.

## Supporting information

S1 Table Number and percentage of missing values in predictor variables. (PDF)

S2 Table Final model fit for LR model, combined with SMOTE method. (PDF)
