# Supplementary material for: Comparative study on the performance of different classification algorithms, combined with pre- and post-processing techniques to handle imbalanced data, in the diagnosis of adult patients with familial hypercholesterolemia
Source: PLoS One. 2022 Jun 24;17(6):e0269713. doi: 10.1371/journal.pone.0269713 (PMC9231719; doi:10.1371/journal.pone.0269713)
Supplement: S1 Table — (PDF) [file pone.0269713.s001.pdf]

S1 Table. Number and percentage of missing values in predictor variables.

	Total		Medicated		non-Medicated	
	FH	non-FH	FH	non-FH	FH	non-FH
Medication	12 (8.2)	27 (8.9)	7 (6.3)	19 (8.5)	5 (14.0)	8 (9.8)
Age	3 (2.0)	4 (1.3)	3 (2.7)	4 (1.8)	-	-
BMI	12 (8.2)	33 (10.8)	7 (6.3)	22 (9.9)	5 (14.0)	11 (13.4)
Lp(a)	10 (6.8)	30 (9.8)	9 (8.1)	20 (9.0)	1 (2.9)	10 (12.2)
ApoAI	2 (1.4)	16 (5.2)	2 (1.8)	10 (4.5)	-	6 (7.3)
ApoB	3 (2.0)	16 (5.2)	3 (2.7)	10 (4.5)	-	6 (7.3)

FH: familial hypercholesterolemia; BMI: body mass index; TC: total cholesterol; LDLc: low density lipoprotein cholesterol; HDLc: high density lipoprotein cholesterol; TG: triglycerides; Lp: lipoprotein; Apo: apolipoprotein.
