# Supplementary material for: Comparative study on the performance of different classification algorithms, combined with pre- and post-processing techniques to handle imbalanced data, in the diagnosis of adult patients with familial hypercholesterolemia
Source: PLoS One. 2022 Jun 24;17(6):e0269713. doi: 10.1371/journal.pone.0269713 (PMC9231719; doi:10.1371/journal.pone.0269713)
Supplement: S2 Table — (PDF) [file pone.0269713.s002.pdf]

S2 Table. Final model fit for LR model, combined with SMOTE method.

	β_j	SE	Wald	p-value	OR	95% CI
(Intercept)	0.95	1.07	0.89	0.38	2.57	(0.32 - 21.25)
Statin Medication	-0.34	0.90	-0.38	0.70	0.71	(0.12 - 4.04)
LDLc	0.03	0.00	10.18	< 0.01	1.03	(1.03 - 1.04)
TG	-0.01	0.00	-5.63	< 0.01	0.99	(0.98 - 0.99)
HDLc	-0.03	0.01	-4.04	< 0.01	0.97	(0.95 - 0.98)
Age	-0.11	0.02	-4.87	< 0.01	0.89	(0.85 - 0.93)
Hypertension	-0.57	0.39	-1.47	0.14	0.57	(0.27 - 1.20)
Physical Signs	0.55	0.34	1.63	0.10	1.74	(0.90 - 3.42)
Smoking	-0.96	0.35	-2.71	0.01	0.38	(0.19 - 0.76)
Male Sex	-0.88	0.28	-3.16	< 0.01	0.42	(0.24 - 0.71)
Hypertension:Male Sex	2.15	0.50	4.28	< 0.01	8.56	(3.24 - 23.23)
Statin Medication:Age	0.08	0.02	3.16	< 0.01	1.08	(1.03 - 1.13)
Hypertension:Smoking	1.18	0.69	1.73	0.08	3.27	(0.87 - 12.95)

SE: standard error; OR: odds ratio; CI: confidence interval; LDLc: low density lipoprotein cholesterol; TG: triglycerides; HDLc: high density lipoprotein cholesterol.
